# Simulating reading acquisition: The link between reading outcome and multimodal brain signatures of letter–speech sound learning in prereaders

**DOI:** 10.1038/s41598-018-24909-8

**Published:** 2018-05-08

**Authors:** Iliana I. Karipidis, Georgette Pleisch, Daniel Brandeis, Alexander Roth, Martina Röthlisberger, Maya Schneebeli, Susanne Walitza, Silvia Brem

**Affiliations:** 1 Department of Child and Adolescent Psychiatry and Psychotherapy, Psychiatric Hospital, University of Zurich, Zurich, Switzerland; 2 University of Zurich and ETH Zurich, Neuroscience Center Zurich, Zurich, Switzerland; 3 Department of Child and Adolescent Psychiatry and Psychotherapy, Central Institute of Mental Health, Medical Faculty Mannheim/Heidelberg University, Mannheim, Germany; 4 Center for Integrative Human Physiology Zurich, University of Zurich, Zurich, Switzerland

## Abstract

During reading acquisition, neural reorganization of the human brain facilitates the integration of letters and speech sounds, which enables successful reading. Neuroimaging and behavioural studies have established that impaired audiovisual integration of letters and speech sounds is a core deficit in individuals with developmental dyslexia. This longitudinal study aimed to identify neural and behavioural markers of audiovisual integration that are related to future reading fluency. We simulated the first step of reading acquisition by performing artificial-letter training with prereading children at risk for dyslexia. Multiple logistic regressions revealed that our training provides new precursors of reading fluency at the beginning of reading acquisition. In addition, an event-related potential around 400 ms and functional magnetic resonance imaging activation patterns in the left planum temporale to audiovisual correspondences improved cross-validated prediction of future poor readers. Finally, an exploratory analysis combining simultaneously acquired electroencephalography and hemodynamic data suggested that modulation of temporoparietal brain regions depended on future reading skills. The multimodal approach demonstrates neural adaptations to audiovisual integration in the developing brain that are related to reading outcome. Despite potential limitations arising from the restricted sample size, our results may have promising implications both for identifying poor-reading children and for monitoring early interventions.

## Introduction

Learning to read involves successfully linking letters with the corresponding speech sounds. Children with developmental dyslexia struggle to learn these associations^1,2^. Behavioural skills offer valuable clues about successful reading acquisition. Phonological awareness, rapid automatized naming (RAN), and letter knowledge have been reported as precursors of future reading success^3–5^, but established behavioural precursors also have clear limitations^1^. Hence, finding more reliable and specific early predictors for dyslexia remains urgent, and a more direct measure of the ability to learn letter–speech sound correspondences could critically improve early identification of children with developmental dyslexia.

To date, neuroimaging studies have provided substantial insights into the emergence of the neural reading circuit^6^. A pivotal part of this circuit specifically develops to integrate audiovisual information. During reading acquisition, posterior parts of the auditory association cortex, the planum temporale (PT) and the superior temporal cortex, adapt to the automatic processing of newly learned associations between letters and speech sounds^7^. In addition, the left ventral occipitotemporal cortex (vOT) develops a specialization for print^8–10^, and a crucial part of this brain region later serves as the visual word form area^11^, facilitating fast and automatic word reading^12^. Therefore, pre-existing neural systems undergo plastic changes to meet the new cognitive demands arising with reading acquisition^9,13^.

Accordingly, deviant neurobiological development of the visual and phonological neural circuits during reading acquisition probably leads to impaired automatization in learning letter–speech sound associations, a core deficit of dyslexic children^14^. Experimental paradigms that manipulate the congruency of letter–speech sound associations enable the operationalization of audiovisual integration^15^. Previous studies have shown that the brain activation difference between congruent and incongruent audiovisual letter–speech sound associations is larger in normal-reading children and adults than in children and adults with developmental dyslexia^16–19^. Thus, the question arises whether neural differentiation of congruent and incongruent information in prereading children relates to their reading development and initial reading skills. Filling this research gap is highly relevant to determining the neurobiological mechanisms that underlie the audiovisual integration deficit at the very early learning stage that precedes reading acquisition.

Combining behavioural and neuroimaging measures can facilitate the identification of future struggling readers, because cortical differentiation of the involved brain networks starts before reading acquisition^20^. So far, several neuroimaging studies aiming to improve prediction of dyslexia have concentrated on comparing prereaders at risk for dyslexia with control subjects^21–23^. Identifying children at risk for dyslexia based on familial history is straightforward. However, familial risk comprises a risk factor with low specificity, given that between 34% and 65% of children at familial risk will eventually develop reading problems^24,25^. We hypothesize that behavioural and neurobiological precursors directly related to the audiovisual integration of letter–speech sound correspondences improve prediction of future reading skills in a high-risk sample and offer a framework for developing novel screening applications for early diagnosis of developmental dyslexia.

To improve early identification of children at risk for dyslexia, we simulated the process of learning letter–speech sound correspondences in prereaders and performed simultaneous electroencephalography (EEG) and functional magnetic resonance imaging (fMRI) recordings during audiovisual integration of the learned correspondences^26^. After half a year of formal reading instruction, the children’s reading fluency was assessed. EEG and fMRI data have been used before to identify early cortical developmental differences predicting later reading success^23,27–30^. Here, we applied simultaneous EEG-fMRI in prereading children. This allowed the advantages of each modality to be exploited and the information gained to be integrated to obtain novel insights on the timing and locus of reading-related neural processes. Our unique child-friendly approach allows the individual ability to learn correspondences to be quantified. It also pinpoints training-induced plastic changes in brain networks adapting to multisensory integration of orthographical and phonological information during reading acquisition. We show that fundamental brain responses during audiovisual integration of single phonological and orthographical units in the prereading stage offer clues regarding reading outcome.

## Results

### Simulation of letter acquisition offers a novel precursor of reading outcome

**Figure 1:**
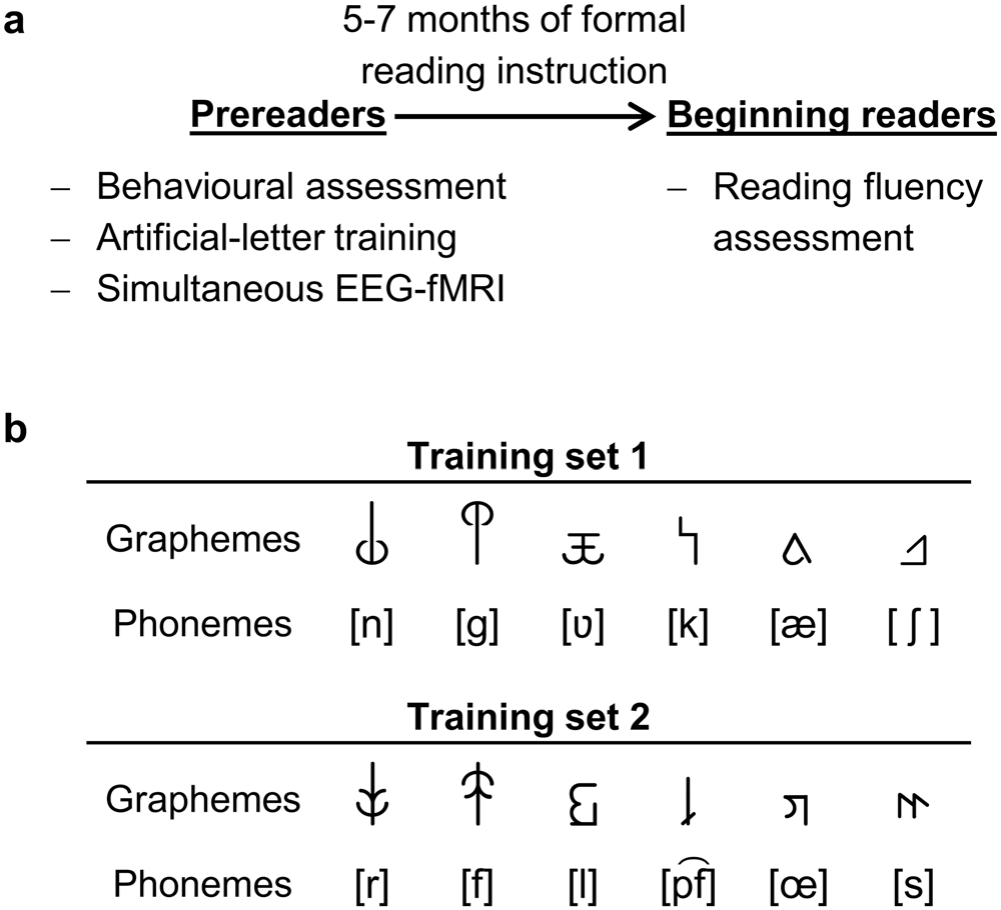
Study design and experimental stimuli. (**a**) In a longitudinal study design, participants were tested before and after the onset of formal reading instruction at school. (**b**) Stimulus sets used in the artificial-letter training and the implicit audiovisual target detection task. Participants were randomly assigned to learn one of two matched sets of grapheme–phoneme correspondences. In a simultaneous EEG-fMRI session, correct (congruent condition) and incorrect (incongruent condition) pairs of trained graphemes and phonemes were presented to the participants. Phonemes are presented in phonetic notation; phonemic notation from left to right for training set 1 is /n/, /g/, /w/, /k/, /ä/, and /sch/ and for training set 2 /r/, /f/, /l/, /pf/, /ö/, and /s/.

In a longitudinal study, 35 prereading children at varying risk for developmental dyslexia were tested for precursor skills of reading, participated in computerized, artificial-letter training, and performed an implicit audiovisual target detection task in a simultaneous EEG-fMRI session during which the trained grapheme–phoneme correspondences were presented (Supplementary Table S1). Here, we analyzed a subsample of 28 subjects (age: 6.7 ± 0.3 years) whose neuroimaging data met stringent quality criteria (Methods). Five to seven months after the onset of formal reading instruction at school, we assessed children’s word and pseudoword reading fluency (age: 7.4 ± 0.3 years). Based on their mean reading fluency, they were classified as children with either normal or poor reading development (Table 1; Fig. 1a).

**Table 1:** Group statistics (*n* = 28).

	Normal readers	Poor readers	Test statistic
Sex (female/male)	6/9	6/7	
Handedness (left/right)	3/12	0/13	
Familial risk for dyslexia^1^	0.54 ± 0.18	0.49 ± 0.14	*t*(26) = −0.76, *P* = 0.608
IQ estimate	109 ± 13	101 ± 14	*t*(26) = 1.53, *P* = 0.336
**Precursor skills of reading**			
Age in years T1	6.7 ± 0.3	6.6 ± 0.4	*t*(26) = 0.87, *P* = 0.608
Phonological awareness^2^	54.0 ± 21.5	40.6 ± 23.0	*t*(26) = 1.60, *P* = 0.336
RAN objects^2^	37.8 ± 19.9	23.6 ± 21.3	*t*(26) = 1.82, *P* = 0.336
letter-speech sound knowledge^3^	15.8 ± 10.6	14.8 ± 11.4	*t*(26) = 0.25, *P* = 0.842
Simple word reading^4^	3.7 ± 5.0	2.5 ± 3.3	*t*(26) = 0.74, *P* = 0.608
Non-word repetition^2^	37.9 ± 23.6	28.3 ± 22.8	*t*(26) = 1.09, *P* = 0.542
Passive vocabulary^2^	61.1 ± 25.6	48.5 ± 29.6	*t*(26) = 1.21, *P* = 0.501
Vocabulary – word meaning^2^	41.5 ± 28.4	58.2 ± 27.2	*t*(26) = −1.58, *P* = 0.336
Training duration in minutes	16.8 ± 4.2	23.2 ± 7.1	*t*(26) = −2.92, *P* = 0.040
Training accuracy in %^5^	80.0 ± 12.0	79.2 ± 7.2	*t*(26) = 0.20, *P* = 0.842
In-scanner task performance in %^6^	91.2 ± 10.1	85.9 ± 16.1	*t*(23) = 1.02, *P* = 0.542
In-scanner reaction time in ms^6^	753 ± 144	711 ± 162	*t*(23) = 0.68, *P* = 0.608
**Initial reading fluency skills**			
Age in years T2	7.4 ± 0.2	7.3 ± 0.4	*t*(26) = 0.67, *P* = 0.702
Word reading fluency^2^	49.4 ± 23.9	6.1 ± 6.5	*t*(26) = 6.33, *P* < 0.001
Pseudoword reading fluency^2^	39.3 ± 24.8	4.3 ± 6.1	*t*(26) = 4.95, *P* < 0.001

Values are mean ± standard deviation, *P*-values are FDR-adjusted. ^1^Highest parental ARHQ score; ^2^percentile scores; ^3^raw values; ^4^number of correctly read one- or two-syllable upper case letter words out of 20; ^5^trial-wise item-weighted accuracy; ^6^*n* = 25, three subjects from the poor reading group excluded (Methods); T1: prereading stage; T2: beginning reading stage.

To determine predictors of reading outcome, we first performed correlations of behavioural reading precursors with reading fluency after half a year of formal reading instruction (Supplementary Table S2). Only RAN correlated significantly with later reading fluency (*r* = 0.574, *P* = 0.0119). No other tested precursor showed a significant relation to reading fluency, including phonological awareness (*r* = 0.354, *P* = 0.1373), letter knowledge (*r* = 0.239, *P* = 0.269), non-word repetition (*r* = 0.171, *P* = 0.436), passive vocabulary (*r* = 0.266, *P* = 0.2431), word meaning (*r* = −0.283, *P* = 0.2224), or the familial risk factor (*r* = −0.008, *P* = 0.9668). This result is in line with previous evidence that RAN is a robust precursor of reading fluency in European languages^31^ and of reading outcome in various alphabetic and non-alphabetic languages^32^.

We also tested for additional precursors of reading based on the simulation of letter acquisition. Using an adaptive computerized training (<40 min), prereaders intensively trained the correspondences of six unknown artificial letters (false-font characters) with six known speech sounds of their native language (German; Fig. 1b). The adaptive nature of the artificial-letter training allowed prereaders’ learning rate and performance to be quantified individually. A higher learning rate, reflected by a shorter training duration and hence faster learning, correlated significantly with initial reading fluency (*r* = −0.678, *P* = 0.0017; Supplementary Fig. S1a). In addition, better performance during the training was marginally related to the children’s initial reading fluency (*r* = 0.398, *P* = 0.0877). Therefore, next to the behavioural precursor RAN, the rate at which prereaders learned artificial letter–speech sound correspondences correlated highly with later reading fluency. Interestingly, the novel precursor derived from the artificial-letter training represents a direct measure of automatization when learning associations between letters and speech sounds; such automatization is a key feature of successful reading acquisition^14,33,34^.

**Figure 2:**
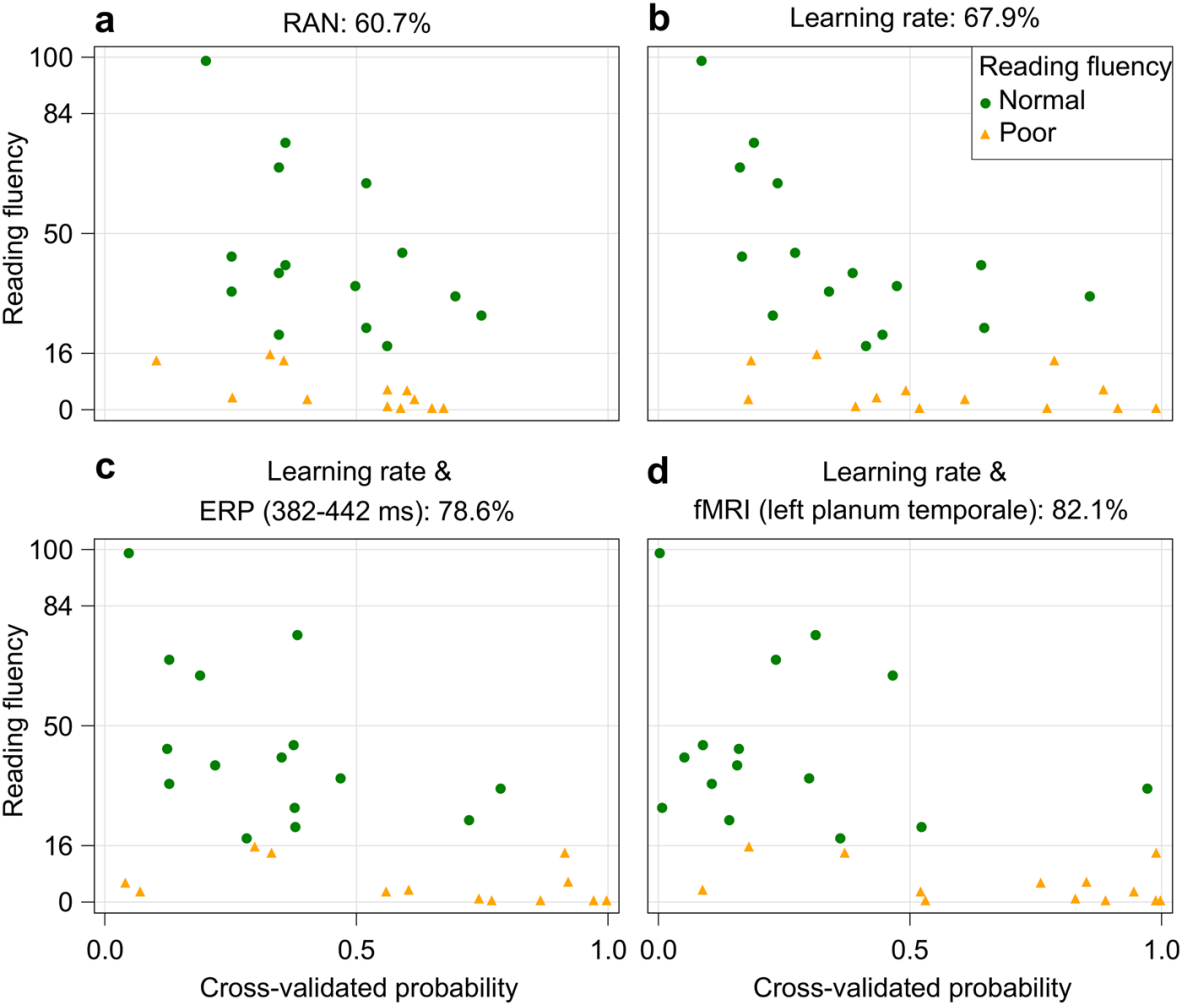
Prediction accuracy of multiple logistic regression models. Each subject’s cross-validated probability of being classified as a poor reader is plotted against each subject’s mean reading fluency (*n* = 28). Factors included in each multiple logistic regression model and overall cross-validated prediction accuracy are superimposed to each plot.

To evaluate the predictive validity of these precursors, we performed multiple logistic regressions with behavioural precursors as independent variables and future reading fluency as the dependent variable. Reading fluency was entered as a nominal variable in the logistic regression model. Children were classified as poor readers if their mean reading fluency was below the 16^th^ percentile (*n* = 13) and as normal readers otherwise (*n* = 15; Table 1)^35^. The behavioural precursor RAN (*P* = 0.0872) predicted later reading fluency with an overall cross-validated accuracy of 60.7% (specificity: 60%; sensitivity: 61.5%; Table 2; Fig. 2a).

**Table 2:** Multiple logistic regression models (*n* = 28).

Parameter	Maximum likelihood estimate	SE	Wald chi-square	*P*-value	Nagelkerke’s pseudo R-square	Sensitivity	Specificity
**Behavioural model**							
Intercept	0.919	0.726	1.603	0.2055	0.148	61.5	60.0
RAN^1^	−0.035	0.020	2.925	0.0872			
Phonological awareness^1^				>0.15			
**Artificial-letter training**							
Intercept	−4.661	2.008	5.390	0.0202	0.344	53.9	80.0
Learning rate^2^	0.232	0.103	5.105	0.0239			
RAN^1^				>0.15			
**Artificial-letter training & left-hemispheric incongruency difference of ERP mean amplitude (382–442 ms)**							
Intercept	−4.508	2.199	4.202	0.0404	0.463	69.2	86.7
Learning rate^2^	0.242	0.116	4.398	0.0360			
ERP (382–442 ms)^3^	−0.491	0.289	2.879	0.0898			
RAN^1^				>0.15			
**Artificial-letter training & incongruency difference of BOLD response in left PT**							
Intercept	−4.267	2.394	3.18	0.0748	0.601	76.9	86.7
BOLD PT^4^	−6.380	2.858	4.98	0.0256			
Learning rate^2^	0.213	0.128	2.79	0.0948			
RAN^1^				>0.15			

^1^Percentile scores; ^2^training duration in minutes; ^3^mean amplitude of incongruency difference over left posterior electrodes of interest at 382–442 ms; ^4^mean beta values of incongruency difference in the left PT ROI; BOLD: blood-oxygen-level dependent, ERP: event-related potential, PT: planum temporale, RAN: rapid automatized naming, SE: standard error.

Adding the learning rate from the artificial-letter training (*P* = 0.0239) to the model improved the overall cross-validated predictive accuracy to 67.9%, meaning that two additional subjects were correctly classified. While this model identified normal readers with higher specificity -80%- its sensitivity of 53.9% in identifying poor readers was low (Table 2; Fig. 2b). Thus, the learning rate in the artificial-letter training not only showed a stronger correlation than RAN with future reading fluency, but also performed better than established behavioural reading precursors in the prediction analysis, especially in the identification of non-affected children. Notably, this result was confirmed in a larger sample in our study which included 35 participants (Supplementary Information, Supplementary Tables S1 and S3, Supplementary Fig. S1b). We also corroborated the finding that the learning rate improved the prediction of reading fluency with a general linear model, which additionally revealed a contribution of phonological awareness (Supplementary Information, Supplementary Table S4).

### Increasing sensitivity with event-related potentials

One to five days after the artificial-letter training, participants performed an audiovisual target detection task^26,36^ during simultaneous EEG and fMRI recordings. The task included the trained grapheme–phoneme correspondences, which were presented audiovisually either as congruent or incongruent pairs (Fig. 1b). We restricted our EEG analyses to two event-related potentials (ERP), which had shown a training-induced modulation of audiovisual activation in an independent previous analysis of a subsample that had not involved future reading performance^26^. For these two ERPs, the comparison between trained grapheme–phoneme correspondences and untrained correspondences had yielded significant differences^26^. In particular, mean amplitude values of ERPs over posterior electrode clusters were calculated for each condition (congruent and incongruent) and each subject for the time windows 382–442 ms (initial window of audiovisual integration) and 644–704 ms (late negativity; Supplementary Fig. S2). The analysis of additional ERP intervals is reported in the Supplementary Information. To test how well these ERPs reflected the effect of audiovisual integration of grapheme–phoneme correspondences on future reading fluency, linear mixed models were calculated with the factors reading fluency (normal vs. poor), congruency (congruent vs. incongruent), and hemisphere (left vs. right).

**Figure 3:**
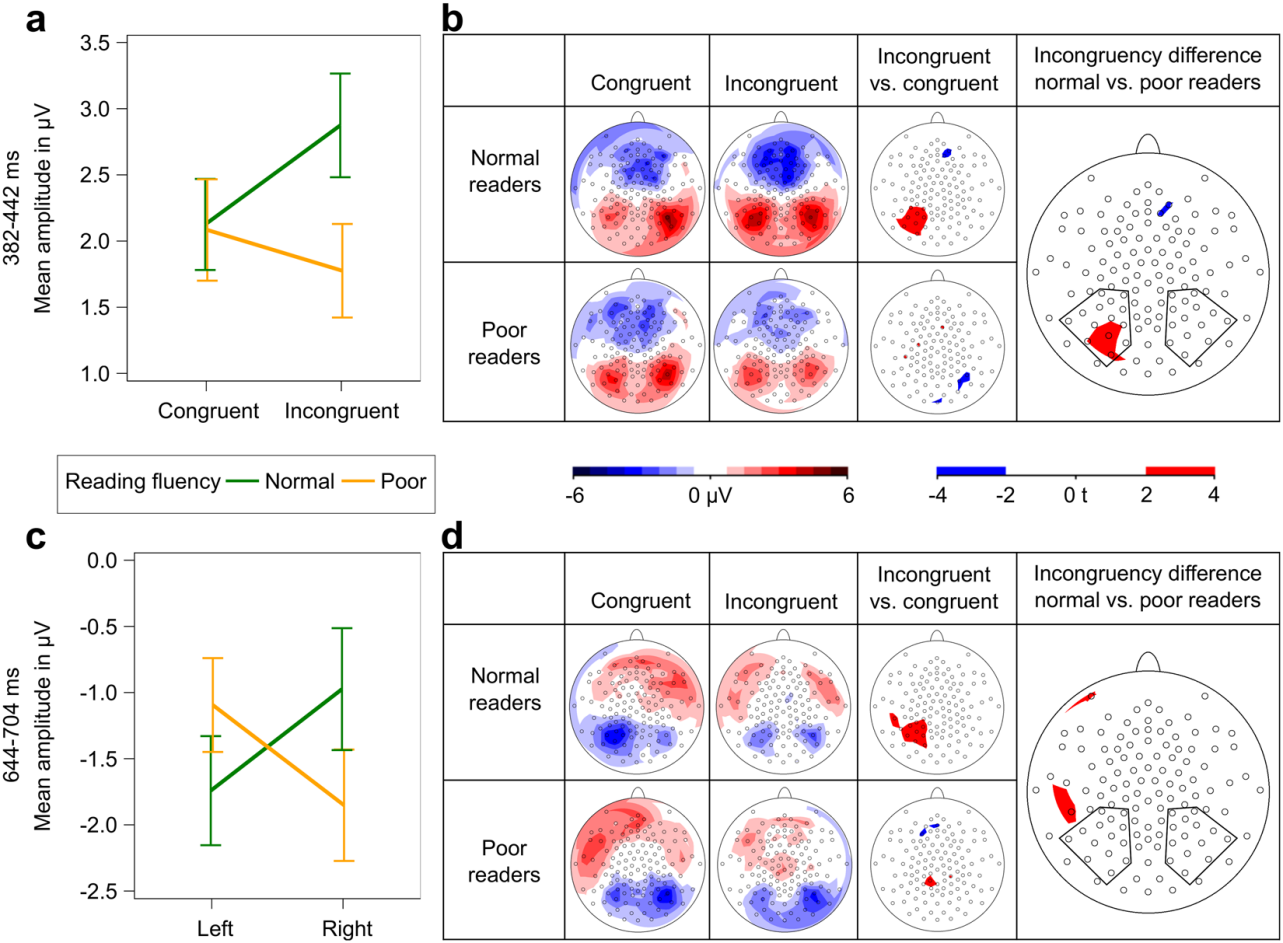
EEG analyses. Mean amplitude values were calculated for electrodes of interest, marked with black polygons (*n* = 28). Error bars illustrate standard error of the mean. (**a**) Significant interaction of congruency and reading fluency for mean amplitude values in the time window 382–442 ms. (**b**) Potential field maps and statistical t-maps of initial ERP of audiovisual integration (382–442 ms). (**c**) Significant interaction of hemisphere and reading fluency for mean amplitude values in the time window 644–704 ms. (**d**) Potential field maps and statistical t-maps of late negativity ERP (644–704 ms).

The initial time window of audiovisual integration (382–442 ms) showed a significant interaction of congruency and reading fluency at posterior electrodes [F(1,77) = 4.87, *P* = 0.0303; Fig. 3a]. Post-hoc t-tests revealed no significant differences between conditions or groups. Calculating the corresponding *t*-maps indicated that this interaction effect seemed to be driven by higher posterior positivity for incongruent pairs than for congruent pairs for future normal readers and was particularly pronounced over the left hemisphere (Fig. 3b). For the late negativity (644–704 ms), the linear mixed model revealed a significant interaction of hemisphere and reading fluency [F(1,78) = 5.94, *P* = 0.0170; Fig. 3c]. Post-hoc t-tests revealed no significant differences between hemispheres or groups. Statistical *t*-maps suggested a hemisphere difference of future normal readers resulting from a reduced left hemispheric negativity for incongruent pairs (Fig. 3d).

Next, we tested whether the reported incongruency effects of the ERP would improve the prediction of reading outcome. We found that the incongruency difference over the left posterior electrodes of the positive deflection (382–442 ms) correlated with future reading fluency on a trend level (*r* = 0.437, *P* = 0.057; Supplementary Fig. S1c). The late negativity ERP (644–704 ms) exhibited no significant correlation effect for the incongruency amplitude differences at the left posterior electrodes (left: *r* = 0.333, *P* = 0.1416; Supplementary Information).

Adding the initial ERP mean amplitude values (382–442 ms), the RAN, and the learning rate to the multiple logistic regression model classified three additional subjects correctly and increased the model’s cross-validated prediction accuracy to 78.6%. The learning rate (*P* = 0.0360) and the ERP incongruency difference (*P* = 0.0898) were included in the model, which resulted in an increase in specificity (86.7%) and sensitivity (69.2%; Table 2; Fig. 2c). Although this finding was not replicated with general linear models (Supplementary Table S4), the left-lateralized posterior positivity around 400 ms may reflect important differences in the neural processes underlying audiovisual integration that might be crucial for successful reading acquisition.

### Audiovisual integration in the left temporal cortex is related to reading outcome

Following a previous analysis of a subsample that determined effects of artificial-letter training^26^, we performed a region of interest analysis (ROI) in a cluster of the right inferior temporal gyrus (ITG). This showed stronger activation when processing trained incongruent audiovisual information than untrained (peak MNI coordinates: x = 43, y = −12, z = −27; for whole-brain analysis see Supplementary Information)^26^. To test the relationship between audiovisual integration in the right ITG and future reading fluency, we calculated a linear mixed model with factors reading fluency (normal vs. poor) and congruency (congruent vs. incongruent). We found a significant main effect of congruency [F(1,26) = 4.81, *P* = 0.0375], which was characterized by stronger hemodynamic responses for incongruent trained pairs than congruent ones (*t*(26) = 2.19); this effect verified our previous results^26^. However, no effect on reading fluency was evident in the right ITG.

**Figure 4:**
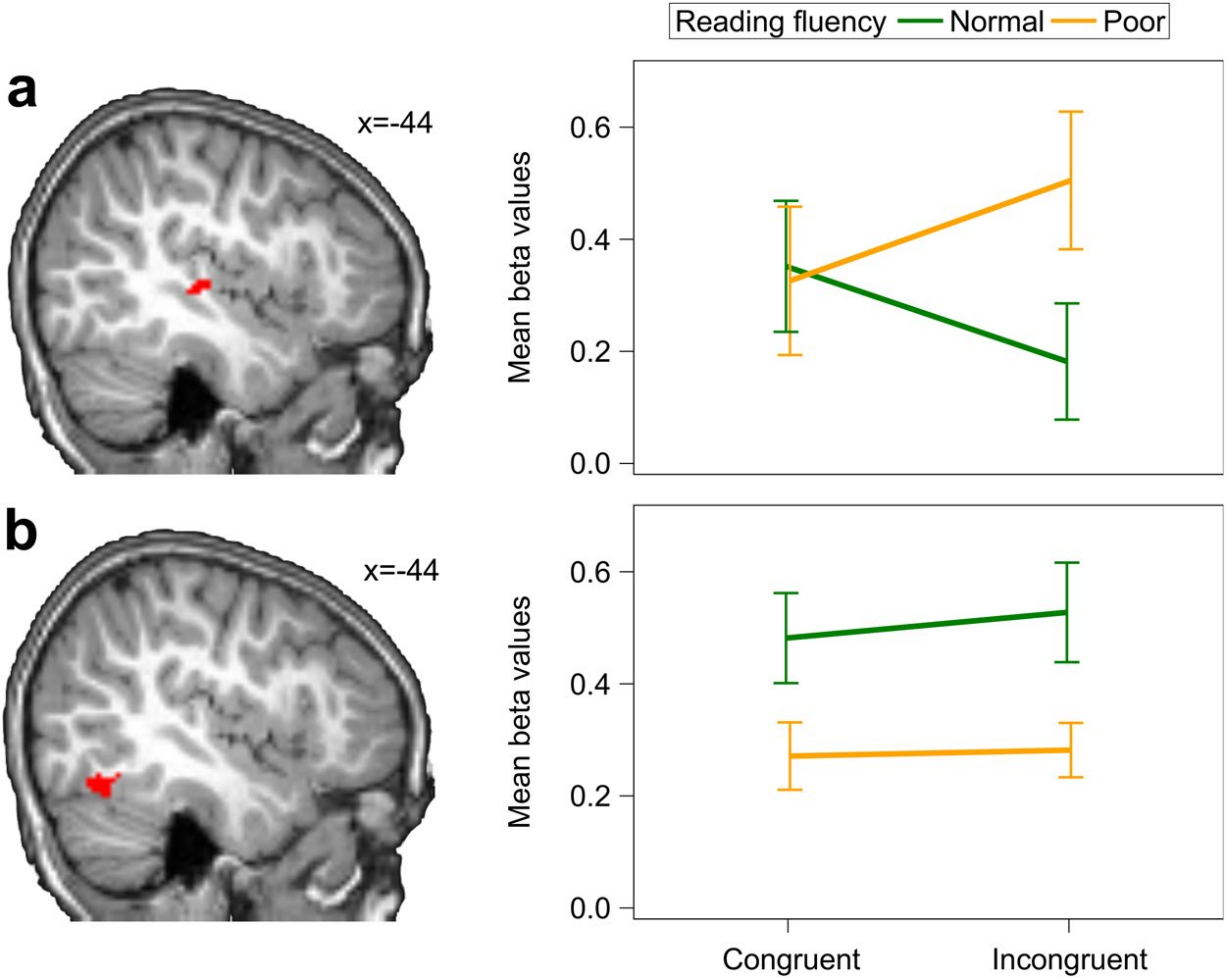
fMRI analyses. Mean beta values were extracted from the region of interests (ROI) projected onto a paediatric structural T1 image normalized to MNI space (*n* = 28). Error bars illustrate standard error of the mean. (**a**) Interaction of congruency and future reading fluency in the left planum temporale (PT). The ROI was defined based on temporoparietal activation over both conditions and groups, and spatially restricted by a 12 mm radius sphere of the PT (center MNI coordinates: x = −44, y = −27, z = 13^18^). (**b**) Main effect of reading fluency in the left ventral occipitotemporal cortex (vOT). The ROI was defined based on occipitotemporal activation over both conditions and groups and spatially restricted by the anatomical boundaries of the fusiform gyrus and a 12 mm radius sphere of the vOT (center MNI coordinates: x = −44, y = −57, z = −15^44^).

To test the hypothesis that functional brain responses in audiovisual integration sites differ between future normal and future poor readers, we performed a ROI analysis in the left PT (see Supplementary Information for ROI analysis in right PT). After extracting mean beta values from the PT ROI, we computed a linear mixed model with factors reading fluency (normal vs. poor) and congruency (congruent vs. incongruent). We found a significant interaction of reading fluency and congruency [F(1,26) = 15.21, *P* = 0.0006; Fig. 4a]. While future normal readers exhibited weaker activation for incongruent pairs than congruent pairs (*t*(26) = −2.79, *P* = 0.0456), future poor readers showed marginally higher activation for incongruent pairs than congruent pairs (*t*(26) = 2.74, *P* = 0.0508). Thus, audiovisual integration of grapheme–phoneme pairs was related to future reading outcome. It has previously been reported that children with normal reading skills exhibit a characteristic congruency effect in the PT when processing letter–speech sound pairs, while in dyslexic children this effect is absent^18^. Here, we showed for the first time that this congruency effect is already present in prereaders who will become normal readers.

In addition to the neural adaptations occurring in the left temporal cortex, learning grapheme–phoneme correspondences has been shown to trigger neural specialization processes in the left vOT^8,37^. To examine the role of this region in audiovisual integration, we extracted mean beta values from the left vOT ROI and computed a linear mixed model with factors reading fluency (normal vs. poor) and congruency (congruent vs. incongruent). In the left vOT ROI, we found a significant main effect of reading fluency [F(1,23) = 6.58, *P* = 0.0173; Fig. 4b], indicating stronger hemodynamic responses for future normal readers than for future poor readers (*t*(23) = 2.56). Thus, future normal readers showed enhanced activation in the left vOT during audiovisual processing of grapheme–phoneme correspondences regardless of congruency (see Supplementary Information for ROI analysis in right vOT).

We also investigated whether vOT and PT activation in prereaders was related to future reading fluency skills. In the left vOT, hemodynamic responses during processing of congruent pairs was correlated with future reading fluency on a trend level (*r* = 0.457, *P* = 0.051), while this correlation was not found for incongruent pairs (*r* = 0.244, *P* = 0.2684). Adding the vOT activation during congruent processing to the behavioural model did not improve the prediction of reading fluency (Supplementary Table S3). The congruency difference in the left PT ROI correlated significantly with future reading fluency (*r* = 0.546, *P* = 0.017; Supplementary Fig. S1d). The model based on the congruency difference in the left PT (*P* = 0.0256) and the learning rate (*P* = 0.0948) resulted in an overall cross-validated prediction accuracy of 82.1% (sensitivity: 76.9%, specificity: 86.7%) for the multiple logistic regression. This led to correct classification of one subject more than the ERP model and achieved the best model fit for the general linear model (Supplementary Table S4). Adding the ERP component to the model did not improve prediction accuracy, suggesting that the two imaging modalities did not capture complementary aspects of audiovisual integration.

### Insights from EEG-informed fMRI analysis

**Figure 5:**
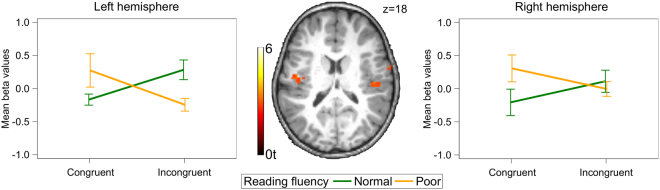
Single-trial EEG-informed fMRI analysis. Mean posterior left-lateralized ERP amplitudes at 382–442 ms modulated hemodynamic responses in left and right temporoparietal brain regions (*n* = 28). Modulation is projected onto a paediatric structural T1 image normalized to MNI space (peak MNI coordinates: left x = −50, y = −12, z = 18; right x = 52, y = −21, z = 12; uncorrected *P* < 0.005, k ≥15). Error bars illustrate standard error of the mean.

To fully exploit the advantages of the simultaneous EEG-fMRI recordings, we performed an exploratory single-trial EEG-informed fMRI whole-brain analysis. In a random-effect generalized linear model (GLM), the hemodynamic responses were trial-wise parameterized by the ERP mean amplitudes of the initial time window of audiovisual integration at 382–442 ms. We then used a 2 × 2 ANOVA to compute the interaction of the factors reading fluency (normal vs. poor) and congruency (congruent vs. incongruent) of this parametric modulation. We found a significant interaction of reading fluency and congruency in bilateral temporoparietal brain regions (Fig. 5, Table 3), bilateral occipital brain regions, and the right anterior cingulate cortex (ACC; Table 3; *P* < 0.005 uncorrected, k ≥ 15). In the left temporoparietal cluster (x = −50, y = −12, z = 18), we found a difference on a trend level between normal and poor readers for the incongruent condition (*t*(26) = 2.41, *P* = 0.0994), but not for the congruent condition (*t*(26) = −2.01, *P* = 0.2099). Differences between normal and poor readers did not reach significance for the right-hemispheric cluster located in the superior temporal gyrus (x = 52, y = −21, z = 12).

**Table 3:** Statistics of EEG-informed fMRI analysis.

Brain area	Hemisphere	MNI coordinates			Voxels	T-value	*P*-value
		x	y	z			
Precentral gyrus	Right	61	−3	12	36	3.70	0.0003
Superior temporal gyrus	Right	52	−21	12	45	3.56	0.0004
Precuneus	Right	34	−78	36	39	3.55	0.0004
Cuneus	Left	−8	−78	9	20	3.55	0.0004
Postcentral gyrus	Left	−50	−12	18	17	3.26	0.0010
Anterior cingulate	Right	16	27	27	20	3.23	0.0011

Voxel-wise uncorrected threshold of *P* < 0.005, k ≥ 15.

Thus, directly incorporating the temporal information of the EEG in the fMRI analysis allowed an association to be inferred between the posterior positive ERP around 400 ms and the activation in temporoparietal brain regions. Despite the lenient statistical threshold used for this exploratory analysis, its result not only converges on the importance of these brain regions for understanding the role of audiovisual integration in reading fluency, but the relation to the ERP data also suggests a specific temporal window during which such integration processes occur. In conclusion, these simultaneous recordings of EEG and fMRI provided novel spatio-temporal insights into neural processes involved in audiovisual integration of single graphemes and phonemes, in which prereaders show functional differences that are related to future success in reading acquisition.

## Discussion

Given that dyslexia is usually diagnosed only after several months or years of formal schooling^6^ with severe psychosocial consequences^38–40^, there is an urgent need for earlier identification of reading problems in beginning readers. We hypothesized that directly tackling the basis and first step in reading acquisition, the ability of prereaders to integrate letter–speech sound correspondences, improves differentiation between normal and poor beginning readers. We showed that early reading fluency in our high-risk sample was related more strongly to artificial-letter training than to established behavioural precursors. The relationship between reading fluency and learning artificial letters was also demonstrated by functional brain differences, reflected in neuroimaging parameters, during audiovisual integration.

This study aimed to examine whether a grapheme–phoneme integration deficit is present in prereading children who will later develop poor reading fluency skills. Despite differences in overall accuracy, all prereading children were able to learn the artificial letter–speech sound correspondences, irrespective of later reading fluency. The main difference between future normal readers and future poor readers was found in the learning rate, a direct measure of learning speed. This result is in line with the finding that dyslexic children perform worse than normal readers when matching graphemes of an artificial-letter script with phonemes under time pressure^41^. Our findings in prereaders provide support for the assumption that impaired reading fluency in dyslexic readers is not caused by the inability to acquire letter–speech sound correspondences per se, but rather by a lack of automatization and fluency in these correspondences^14,33,34^.

The neuroimaging data of prereading children presented here facilitate a novel understanding of the neurobiological mechanisms underlying this audiovisual automatization deficit before reading acquisition. In a previous publication, we showed that prereaders engaged the same brain regions for processing trained artificial letter–speech sound correspondences that will later become part of the reading network^26^. Here, we extend this finding by demonstrating that prereading differences in audiovisual integration are significantly related to reading fluency at the start of reading acquisition. Prereaders who later developed normal reading fluency skills tend to show an enhanced posterior positivity around 400 ms for incongruent audiovisual stimuli; this may reflect the detection of a mismatch between the auditory and visual information^42^. The results of our exploratory EEG-informed fMRI analysis might explain this sensitivity for mismatch detection in future normal readers by a recruitment of temporoparietal brain regions, occipital brain regions, and the ACC. However, given the limitation of a lenient, uncorrected threshold applied for this specific analysis, this interpretation must clearly remain tentative. Nevertheless, electrophysiological audiovisual mismatch sensitivity has been proposed to be related to automatized integration processes occurring in temporoparietal brain regions^43^. Interestingly, in previous analyses, the ERP component and hemodynamic responses in temporal and parietal brain regions were significantly related to performance during artificial-letter training^26^.

The phonological and orthographical deficit theories, which highlight the role of the left-hemispheric brain regions in reading development^44^, led us to expect a left-lateralized modulation of the temporal cortex. A targeted analysis of the hemodynamic responses in the left temporoparietal and occipitotemporal regions of the neural reading network revealed that deviations in neural functioning of these key regions are already present before reading acquisition. Future poor readers showed lower activation in the left vOT when processing the trained audiovisual stimuli. Given that no effect of congruency was found, it remains to be clarified whether a reduced engagement of the left vOT is a general precursor for reading impairment or whether it represents a lack of visual specialization, which has been shown to emerge quickly following short script training^8,45–47^.

We identified training-specific audiovisual integration patterns in the temporoparietal reading network that were related to later reading fluency skills. Future normal readers showed lower activation in the left PT for incongruent pairs than for congruent pairs, while future poor readers showed only a trend, and for the opposite pattern. This effect in the left PT is in line with studies reporting stronger activation for congruent letter-speech sound correspondences than incongruent ones in the temporal cortices of Dutch normal-reading adults and children, but not of dyslexics^16,18^. The direction of the congruency sensitivity differed in the PT from the one found in the ERP data, but the effect in the PT was most likely also driven by the difference in processing incongruent artificial letter–speech sound pairs between children with normal and poor reading development. However, further evidence is needed to confirm this. The left superior temporal cortex has been discussed as the main site of audiovisual integration in the brain’s functional reading network^7,33^ and it has been reported that dyslexic readers demonstrate reduced neuronal responses in this region during integration of letters and speech sounds^16,18^. In agreement with this account, we show that an audiovisual integration deviation in the left PT is already evident in prereaders after a short training.

The application of artificial-letter training revealed emerging audiovisual integration processes in prereaders at risk for dyslexia, and these provided novel precursors of early reading fluency outcome. However, conclusions cannot be drawn from the present sample regarding the role of audiovisual integration in the reading development of children not at risk for dyslexia. Future studies should investigate whether functional brain differences in the form of diminished congruency sensitivity during audiovisual integration are also evident in children who are not at risk for dyslexia but who will nonetheless show poor reading development.

In addition, the limited sample size did not allow the role of different risk profiles to be considered; this represents a limitation for the generalizability and interpretation of prediction results and the EEG-informed fMRI analysis. The prediction accuracy of the behavioural and neuroimaging data of this study needs urgently to be verified by replication studies on larger samples of prereading children. Moreover, a detailed investigation of differential risk profiles and reading disorder profiles in a larger sample seems highly relevant to determining the role of letter–speech sound integration in reading acquisition and to validating the predictive measures. Understanding the neurobiological differences between children with varying risk profiles and their relation to various subtypes of dyslexia will have important implications for its early and effective diagnosis.

Furthermore, the present study investigated reading fluency at a very early learning stage, after only half a year of formal reading instruction. It can be assumed that differentiating between normal-reading children and poor-reading children is difficult at such an early learning stage; developmental dyslexia is usually diagnosed later. This may explain the weaker relationships in this study between early reading fluency and such typical behavioural reading precursors as phonological awareness and letter knowledge than in studies that used reading measures at more advanced learning stages^3,4,24,28,48,49^.

An additional factor that may have affected measuring reading fluency is that diverse teaching methods are used in local schools from which our sample was drawn, and these may have influenced reading development. A longitudinal assessment of children’s reading skills extending to the middle of second grade or later would clarify the developmental trajectories of children’s reading acquisition and would enable comparisons between early reading fluency and reading skills at a more consolidated stage of reading development. Although measuring reading fluency at the beginning of reading acquisition is associated with challenges, investigating early reading development and its relationship with behavioural and neurobiological factors is highly relevant to improving the early identification of children at risk for dyslexia with poor reading outcome.

These novel insights about the neurobiological basis of letter–speech sound association learning in prereading children not only enable the development of new applications for improving early identification; they also have important implications for the design of appropriate interventions. We provide evidence that the audiovisual integration deficit is already apparent before reading acquisition. Hence, developing test instruments that directly assess the ability to associate letters with speech sounds could significantly improve early identification of poor-reading children. Additionally, it has recently been shown that assessments using artificial-letter training can predict the outcome of reading interventions^50^. Thus, the simulation of letter acquisition in prereaders could indicate which children with an audiovisual integration deficit would benefit most from an early intervention. Based on recent behavioural and neurophysiological evidence^51,52^, early interventions aiming to improve the reading fluency of poor readers would benefit from focusing on the automatization of letter–speech sound associations.

In summary, we demonstrate the potential predictive value of artificial-letter training and show that its neural underpinnings in prereaders are related to early reading fluency skills. We provide evidence that the temporal cortex plays a crucial role in audiovisual integration even before reading acquisition begins and that training-induced adaptations in this key region are significantly related to reading outcome at an early stage. By simultaneously combining two non-invasive neuroimaging methods, we identified an ERP of initial audiovisual integration around 400 ms as a possible electrophysiological counterpart of the audiovisual integration processes occurring in the temporoparietal neural circuit of prereaders. Identifying audiovisual integration deficits before the onset of formal reading instruction has potential implications for targeted prevention and intervention programs. In conclusion, combining behavioural and multimodal neuroimaging data provides a novel framework for studying young populations and allows the current understanding of neurodevelopmental disorders, such as dyslexia, to be refined.

## Methods

### Participants

In this longitudinal study, we tested 35 German-speaking children (Supplementary Table S1). Training-related congruency effects of a subsample of 20 children, have already been analyzed and reported^26^. All children were at varying risk for developmental dyslexia, as indicated by their parents in the Adult Reading History Questionnaire (ARHQ^53^; Supplementary Information). Participants had no visual or auditory impairment, no neurological or psychiatric diagnosis, and had a non-verbal IQ-estimate ≥ 80. Participants and their parents gave oral and written informed consent to participate respectively. The study was approved by the research ethics committee of the canton of Zurich and neighbouring cantons in Switzerland. All experiments were performed in accordance with relevant guidelines and regulations of the approving local ethics committee. Seven children were excluded from EEG and fMRI analyses because they did not meet the stringent data quality criteria. The main analyses included the remaining 28 children (Table 1).

### Assessment of prereading skills

Prereading skills were screened with an extensive behavioural test battery in a four months period before the onset of formal reading instruction. The behavioural assessment included an IQ estimate (subtest “block design” of the Wechsler Intelligence Scale for Children^54^), phonological awareness skills^55^, RAN of objects^55^, letter-speech sound knowledge of all lower and upper case letters (52 in total), phonological processing based on non-word repetition^56^, passive vocabulary and word meaning^57^. We assessed initial reading skills of kindergarten children with a word reading test, including 20 simple one- or two-syllable upper case letter words. Children’s mean performance in the reading test suggested that only some children were able to correctly read a few simple short words before school enrolment (*n* = 35: 2.9 ± 4.0 words/*n* = 28: 3.2 ± 4.2 words); therefore, the sample consisted of prereaders.

### Assessment of reading fluency

After five to seven months of formal reading instruction at school, all children came back to perform 1-minute word and pseudoword reading fluency tests (SLRT-II^58^). A cut-off of one standard deviation from the mean^35^ was applied to standardized mean word and pseudoword reading fluency scores (Supplementary Information) to define whether children had normal or poor reading fluency skills (*n* = 35: 17 poor readers/*n* = 28: 13 poor readers).

### Artificial-letter training

We simulated the process of learning letter-speech sound correspondences, using an artificial script the children had no previous exposure to. Based on six letters of the Swiss School Font (b, d, m, t, u, z) two matched false font sets were created, and children were assigned to train the associations of one false font set with speech sounds of the German language (Fig. 1). Adaptive randomization was used to ensure a balanced assignment to the two sets and to control for sex (set 1: *n* = 18, 9 females, 8 poor readers; set 2: *n* = 17, 7 females, 9 poor readers). For the main sample (*n* = 28), 14 participants were assigned to train each false font set (set 1: 6 females, 6 poor readers; set 2: 6 females, 7 poor readers). To control for effects of visual familiarity, false fonts from the untrained set were implicitly presented throughout the training.

The artificial-letter training was performed, using the computer-based training platform GraphoGame^26,59,60^ (Supplementary Information). Due to the adaptive design of the training, items with high error rates were trained more extensively in additional trials (*n* = 35: 82.9 ± 37.2, range: 15–172 trials; *n* = 28: 88.0 ± 38.9, range 15–172 trials). Thus, not only response accuracy but also training duration varied between participants. Children who learned the correspondences fast had low error rates, and thus a high learning rate, reflected by a decreased training duration. In contrast, children who needed more time to learn the correspondences had high error rates, and therefore a low learning rate, which was reflected by an increased training duration. On the day of the simultaneous EEG-fMRI recordings the learned correspondences were repeated in a short training session (*n* = 35: 5.07 ± 1.01 min; *n* = 28: 4.98 ± 1.00 min).

### Experimental paradigm

Participants solved an implicit audiovisual target detection task^26,36^ during simultaneous EEG-fMRI recordings that were performed 2.3 ± 1.3 days after the training and 12.3 ± 9.7 days after the assessment of prereading skills. Using Presentation® (Version 16.4, www.neurobs.com), participants were presented with congruent and incongruent pairs of the trained grapheme-phoneme correspondences (Supplementary Information). A drawing of a turtle, a bell chime, and an audiovisual presentation of both were used as targets, to which participants were instructed to respond by button press (Supplementary Information).

### Simultaneous EEG-fMRI: acquisition parameters

EEG recordings were performed with an MR-compatible high-density EEG system (Net Amps 400, 128-channel EGI HydroCel Geodesic Sensor Net). The Cz electrode served as recording reference and an electrode posterior to Cz as ground. Impedances were kept below 50 kΩ. An electrocardiogram and EEG data were collected with a sampling rate of 1 kHz using a DC-filter and was synchronized to the MRI scanner clock for adequate correction of gradient artefacts^61,62^. For ERP analysis, data were preprocessed (Supplementary Information), epoched from 50 ms prior to 800 ms after stimulus onset, and rereferenced to the average reference^63^. Epochs were averaged for each subject for the bimodal congruent and incongruent conditions, using a cut-off of 15 epochs per condition (40.07 ± 8.63 epochs, range: 19–54).

MRI data was recorded on a Philips Achieva 3 Tesla scanner (Best, The Netherlands) using a 32-element receive head coil. We acquired 189 volumes using a T2*-weighted whole-brain gradient-echo planar image sequence. The following acquisition parameters were used: slices/volume: 31, repetition time: 1.98 s, echo time: 30 ms, slice thickness: 3.5 mm, slice gap: 0.5 mm, flip angle: 80°, field of view: 240 × 240 mm^2^, in-plane resolution: 3 × 3 mm^2^, SofTone factor: 3, sensitivity-encoding (SENSE) reduction factor: 2.2. A field map and a high-resolution T1-weighted anatomical image were acquired for each participant. For fMRI data preprocessing and statistical analysis SPM12 was used (Supplementary Information). Data sets with more than 10% of the scans exceeding a scan-to-scan motion threshold of 1.5 mm/TR or with overall motion above 6 mm were excluded from further analyses. Movement artefacts in the remaining data sets were corrected using the ArtRepair toolbox^64^ (Supplementary Information).

### ERP analysis

Two time windows were defined for ERP analysis based on previous findings in a subsample of this study^26^. Mean amplitude values of posterior electrode clusters (Supplementary Information) were calculated for each condition for the intervals 382–442 ms and 644–704 ms. Statistical analyses were performed with SAS 9.4 (SAS Institute, Cary NC) by calculating linear mixed models (LMM) including the random intercept of each subject and the fixed factors hemisphere (left vs. right), congruency (incongruent vs. congruent), and reading fluency (normal vs. poor). Outliers were identified based on standardized residuals and data points with values below −3 and above 3 were excluded from further analyses^26,65^. Inspection of QQ-plots and predicted versus residual plots ensured that the assumptions of normality and homoscedasticity were met respectively. In case of significant interactions, we performed post hoc t-tests and only report *P*-values corrected for multiple comparisons using the Tukey-Kramer method. As common in ERP and neuroimaging studies, a priori defined ERP intervals and electrode clusters of interest were used to identify group differences in audiovisual integration and no correction was performed regarding multiple comparisons of time windows and number of electrodes.

### fMRI analysis

For each participant, a random-effect generalized linear model (GLM) was calculated, including all conditions (Supplementary Information) and six movement parameters. Focusing on the audiovisual conditions, a 2 × 2 analysis of variance (ANOVA) was performed to test the interaction of the factors congruency (incongruent vs. congruent) and reading fluency (normal vs. poor). A cluster-based family-wise error corrected (FWE-corr) threshold of *P* < 0.05 was applied on a voxel-wise uncorrected threshold of *P* < 0.001.

### ROI analysis

Two ROIs were defined in the left planum temporale (PT) and the ventral occipitotemporal cortex (vOT), using MarsBaR^66^ and the Talairach Daemon (TD) database^67^ (WFU Pickatlas, version 2.4^68^; Supplementary Information). For both ROIs, mean beta values were extracted and linear mixed models with the factors congruency (incongruent vs. congruent) and reading fluency (normal vs. poor) were performed. For outlier exclusion, data inspection, and post-hoc comparisons we used the same procedure as for ERP data.

### EEG-informed fMRI analysis

To investigate how posterior left-lateralized ERP amplitudes of the time window 382–442 ms modulated hemodynamic responses, an additional GLM was defined. Next to the six predictors and movement parameters included in the fMRI analysis, two parametric modulators were used for the audiovisual congruent and incongruent condition and an additional regressor of no interest was introduced to model trials with insufficient EEG quality. The ERP amplitudes used as parametric modulators from the left posterior electrode cluster were trial-wise extracted^69^ and z-transformed within each participant over both conditions before being entered to the model. With a 2 × 2 analysis of variance (ANOVA) parametric modulation was tested for the interaction of the factors congruency (incongruent vs. congruent) and reading fluency (normal vs. poor). For this exploratory analysis, a voxel-wise uncorrected threshold of *P* < 0.005 with a minimal cluster extent of k ≥ 15 was used.

### Prediction analysis

To identify variables suited for prediction analysis, correlational analyses of reading fluency scores and prereading data were performed using SPSS (Version 23.0, Armonk, NY: IBM Corp.). Performing the Shapiro-Wilk test revealed that reading fluency scores were not normally distributed (*n* = 35: *P* = 0.001; *n* = 28: *P* = 0.003), and therefore non-parametric two-tailed Spearman correlations were calculated. Additionally, reading fluency groups were compared with two-tailed independent samples t-tests. *P*-values were corrected for multiple comparisons using the false discovery rate (FDR) approach^70^.

Multiple logistic regressions were calculated with SAS 9.4 (SAS Institute, Cary NC). Parameter estimates of the logistic response function were calculated using the maximum likelihood method as implemented in the PROC LOGISTIC process (Fisher estimation technique). To select independent variables with the highest predictive influence on the dependent variable (reading fluency) a stepwise forward selection procedure was used. Independent variables with *P* < 0.15 were entered and stayed in the regression model. To evaluate model fit of the model including the selected independent variables, Nagelkerke’s pseudo R-square values were calculated and Hosmer and Lemeshow goodness of fit test was performed. To avoid overfitting, a maximum of three factors were entered for selection into the model. Model sensitivity and specificity were computed using the leave-one-out cross-validation method.

### Data availability

The data that support the findings of this study are available from the corresponding author upon reasonable request. The data are not publicly available due to restricted consent of research participants. The artificial-letter training was developed at the Department of Child and Adolescent Psychiatry and Psychotherapy, Psychiatric Hospital, University of Zurich using the GraphoGame platform provided by the University Jyväskylä and is subjected to copyright.

## Electronic supplementary material

Supplementary Information
