# Supplementary material for: Simulating reading acquisition: The link between reading outcome and multimodal brain signatures of letter–speech sound learning in prereaders
Source: Sci Rep. 2018 May 8;8:7121. doi: 10.1038/s41598-018-24909-8 (PMC5940897; doi:10.1038/s41598-018-24909-8)
Supplement: Supplementary file 1 — Supplementary Information [file 41598_2018_24909_MOESM1_ESM.pdf]

SUPPLEMENTARY INFORMATION

Simulating reading acquisition: The link between reading outcome and multimodal brain signatures of letter–speech sound learning in prereaders

Authors: Iliana I. Karipidis[1,2], Georgette Pleisch[1,2], Daniel Brandeis[1,2,3,4], Alexander Roth[1], Martina Röthlisberger[1], Maya Schneebeli[1], Susanne Walitza[1,2,4], Silvia Brem*[1,2]

[1]Department of Child and Adolescent Psychiatry and Psychotherapy, Psychiatric Hospital, University of Zurich, Switzerland.

[2]University of Zurich and ETH Zurich, Neuroscience Center Zurich, Switzerland.

[3]Department of Child and Adolescent Psychiatry and Psychotherapy, Central Institute of Mental Health, Medical Faculty Mannheim/Heidelberg University, Mannheim, Germany.

[4]Center for Integrative Human Physiology Zurich, University of Zurich, Switzerland.

Corresponding author:

*Silvia Brem, Neumuensterallee 9, CH-8032 Zurich, Switzerland, sbrem@kjpd.uzh.ch.

Replication of prediction based on artificial-letter training in enlarged sample

To confirm the higher predictive accuracy of training-based learning rate compared with RAN, the prediction analysis was also performed in a larger sample including 35 participants (18 normal, 17 poor; Supplementary Table S1 and S3). Again, the cross-validated prediction accuracy of the learning rate in the artificial-letter training (*P*=0.0210; 68.6%; sensitivity: 58.8%; specificity: 77.8%) outperformed the established behavioural reading precursors (RAN *P*=0.1109; 57.1%; sensitivity: 64.7%; specificity: 50%). The comparable results in this larger sample reinforce the finding of the manuscript.

Correlation of working memory and artificial-letter training parameters

To investigate the relationship of working memory and artificial-letter training parameters, we performed additional correlational analyses. We found no significant correlation of non-word repetition with neither training duration (*n*=28: Spearman's *r* = -0.29 *P*=0.268/ *n*=35 Spearman's *r* = -0.327 *P*=0.110) nor weighted training accuracy (*n*=28: Pearson's *r* = 0.056 *P*=0.776/ *n*=35: Pearson's *r* = -0.059 *P*=0.738).

Prediction based on late negativity ERP

Adding the left-hemispheric incongruency difference of the late negativity (644-704 ms), RAN, and the learning rate to the multiple logistic regression model resulted in a somewhat reduced predictive cross-validated accuracy of 75% (Supplementary Table S3). Based on the applied stepwise procedure, the late negativity ERP (*P*=0.0747) and the learning rate (*P*=0.0261) were included in the model (specificity: 80%; sensitivity: 69.2%).

This result confirms that ERP data reflecting audiovisual integration improve the prediction of future reading outcome. Including the ERP of the initial time window of audiovisual integration (382-442 ms) to the model resulted in a better prediction than including the late negativity ERP (644-704 ms). Including both ERP components (382-442 ms: *P*=0.0986; 644-704 ms: *P*=0.0769), along with the learning rate (*P*=0.0290), in the multiple logistic

regression model only reached a cross-validated prediction accuracy of 71.4%, which was lower that the prediction accuracy of the models including each ERP component separately (Supplementary Table S3). Therefore, the two ERP components probably reflect distinct neural processes, each of which carries different information regarding audiovisual integration that might be crucial for successful reading acquisition.

Supplementary ERP analyses

Time windows were selected using adaptive segmentation based on global field power (GFP) peaks to define ERP components[1]. We calculated the GFP of the mean ERPs over all conditions and identified 4 peaks at 142, 232, 356, and 674 ms corresponding to the P1, N1, P400, and late negativity respectively. Time windows were defined as the interval +/-30 ms around the GFP peaks (112-172 ms, 202-262 ms, 326-386 ms, and 644-704 ms). Because the third peak (356 ms) was a longer lasting component, two additional time windows of 60 ms were defined subsequently (386-446 ms and 446-506 ms). Mean amplitude values of posterior electrode clusters were calculated for each condition for the time windows 112-172 ms, 202-262 ms, 326-386 ms, 386-446 ms, and 446-506 ms, but not for the time window 644-704 ms, because it is reported in the main analysis.

Linear mixed models with the factors reading fluency (normal vs. poor), congruency (congruent vs. incongruent), and hemisphere (left vs. right) were calculated. For the time windows 112-172 ms and 202-262 ms the linear mixed model showed no significant effect. The linear mixed model with the mean amplitude values of the time window 326-386 ms revealed a significant main effect of hemisphere with a stronger positivity over the right hemisphere than the left hemisphere [$F(1,77)=4.69$, $P=0.0335$, $t=2.17$]. Significant interactions of the factors congruency and reading were found for the time windows 386-446 [$F(1,77)=5.34$, $P=0.0235$] and 446-506 [$F(1,77)=5.76$, $P=0.0188$].

Supplementary fMRI analysis

Event-related BOLD activity was analyzed by computing a 2 x 2 analysis of variance (ANOVA) to investigate the interaction of the factors reading fluency (normal vs. poor) and congruency (incongruent vs. congruent). Whole-brain analysis revealed a significant interaction of reading fluency and congruency in the right middle frontal gyrus (MFG; $P<0.001$ uncorrected, cluster level corrected $P<0.05$; Supplementary Figure S3). While future poor readers engaged the right MFG significantly stronger for incongruent than congruent pairs ($t(26)=3.51$, $P=0.0084$), future normal readers showed a significantly stronger deactivation for incongruent than congruent pairs ($t(26)=-3.17$, $P=0.0189$) in this region. For incongruent pairs, activation in the right MFG was significantly enhanced for poor readers compared with normal readers ($t(26)=4.43$, $P=0.0008$), while no difference was found for the congruent condition ($t(26)=-1.47$, $P=0.4716$). Hence, the incongruency difference, was significantly more positive for future poor readers than normal readers ($t(26)=4.73$, $P<0.0001$). In beginning readers, an overactivation in the right MFG has been previously reported to predict poor word decoding skills[2], an ability that strongly relies on letter-speech sound binding.

Linear mixed models with the factors reading fluency (normal vs. poor) and congruency (congruent vs. incongruent) were also performed for mean beta values derived from the right hemispheric PT and vOT ROIs. These analyses yielded no significant main or interaction effects.

Methods

Familial risk for dyslexia: Parents completed the Adult Reading History Questionnaire (ARHQ) to assess familial risk for dyslexia. 31 children had at least one parent with an ARHQ value greater than 0.3, indicating an increased familial risk, 2 children had an older sibling with reading difficulties, and 2 children had a history of specific language impairment.

Standardization of reading fluency test: Due to the lack of age-matched norms in the middle of 1st grade, the fluency tests were standardized in an independent sample of 75 German-speaking children, coming from similar school districts as the sample of this study. With parents' written informed consent, test standardization was performed at school, also five to seven months after the onset of formal reading acquisition.

Artificial-letter training: First, participants were introduced to the six grapheme-phoneme correspondences, while each false font character appeared on a computer screen and its corresponding speech sound was presented over headphones. Then, in a series of test trials, participants were presented with one speech sound and two to four false font characters with the instruction to click on the false font character that was previously associated with this speech sound. The trials of the computerized training contained background images including a banner on which one to six false font characters of the untrained set were shown implicitly but clearly. Participants' performance was calculated based on 131 test trials by introducing a weighting factor, accounting for the varying number of presented items per trial ($Aw = I/Imax*A$; Aw: weighted accuracy, I: number of presented items; $Imax$: maximum permissible number of presented items, A: unweighted accuracy of the trial – correct/ incorrect).

Task design: The task included block-wise presentation of bimodal congruent and incongruent, and unimodal visual and auditory stimuli (four conditions). In each condition, the six trained false fonts and/or speech sounds were presented 9 times, resulting in 54 trials per condition. Stimulus presentation time was 613 ms with an interstimulus interval of 331 or 695 ms. 16 unimodal and bimodal blocks (four blocks per condition) alternated pseudorandomly and consisted of 15 randomly presented stimuli and targets (6 targets per condition). Fixation periods of 6 or 12 s were presented between blocks. The duration of the task was 375 s. Next to the described paradigm, the subjects also completed three further parts

of the implicit audiovisual target detection task, including untrained false fonts and phonemes, real letters and the corresponding speech sounds, and digits and spoken number names.

Presentation of stimuli: Using video goggles (VisuaStimDigital, Resonance Technology, Northridge, CA), false font characters were centrally presented in black on a grey background (mean visual angle: 2.8º horizontally; 4.8º vertically). Speech sounds, spoken by a female speaker, were digitally recorded (sampling rate: 44.1 kHz; 32 bit) and normalized using Audacity (± 1 dB). For high-quality binaural auditory stimulation, in-ear headphones were used (MR confon GmbH, Magdeburg) and acoustic noise of the MRI was kept to the minimum, by implementing a SofTone factor in the sequence. Participants wore sound-absorbing over-ear headphones, which were additionally padded with a custom made foam layer. Sound level was individually adjusted. In addition, a sound-absorbing mat was placed around the participants in the MRI bore. Next to conventional head-padding, we used a custom-made EEG head pad to reduce head movement.

Task performance: Mean task performance and reaction times were calculated for each participant. Group comparisons (normal vs. poor reading fluency) of mean task performance and reaction times were performed with two-tailed independent t-tests. Three subjects from the poor reading group were excluded from this analysis due to technical problems, resulting in inaccurate task performance recording.

EEG data processing: EEG data processing was performed using Brain Vision Analyzer (Version: 2.0; Brainproducts GmbH, Munich, Germany). First, bad channels were interpolated (2.5±1.8, range 0–6 channels), followed by MR gradient (average template subtraction method[3]) and cardioballistic artefact correction (sliding average template subtraction). Then, EEG data were bandpass filtered at 0.1–30 Hz and 50 Hz noise removal (notch) was applied before downsampling to 500 Hz. Independent component analysis (ICA) was performed to correct for eye movements and residual cardioballistic artefacts. Residual

artefacts were manually excluded and intervals with amplitudes ranging from -200 µV to 200 µV were discarded. Four artefact-affected cheek electrodes were excluded from statistical analyses (E43, E48, E119, E120).

fMRI data preprocessing: Preprocessing steps were performed in the following order: B0 field map correction, realignment and unwarping, slice time correction, coregistration and segmentation, normalization, resampling (3 x 3 x 3 mm^3) and smoothing (6 mm FWHM). Normalization to Montreal Neurological Institute (MNI) standard space was performed based on deformations derived from the segmentation and a paediatric anatomical template (age range 5.9–8.5 years) created using the Template-OMatic toolbox[4].

Movement artefact correction: Movement artefact correction was performed using the ArtRepair toolbox[5]. Based on the scan-to-scan motion threshold of 1.5 mm/TR, affected volumes were repaired using linear interpolation between the nearest unrepaired scans. Out of 28 data sets, 12 had at least one volume exceeding the defined threshold and less than 8.5% of the scans were repaired per participant. The data sets of four participants were not analyzed in full length either because the last blocks exceeded the defined motion thresholds (2 subjects, 4 and 6 blocks discarded respectively) or because scanning had to be stopped prematurely (2 subjects, 2 blocks discarded).

Definition of electrode clusters: Posterior electrode clusters included 14 electrodes of interest over each hemisphere (left: E47, E50, E51, E52 [=P3], E53, E57, E58 [=T5], E59, E60, E64, E65, E66, E69, E70 [=O1]; right: E83 [=O2], E84, E85, E86, E89, E90, E91, E92 [=P4], E95, E96 [=T6], E97, E98, E100, E101).

Definition of ROIs: For the PT ROI, a functional mask was created, including the sum (logical OR) of the parietotemporal activations across conditions (congruent and incongruent) and groups (normal and poor reading fluency) on a cluster-based family-wise error corrected

(FWE-corr) threshold of $P<0.05$ (voxel-wise uncorrected $P<0.001$). Then, we generated a 12 mm radius sphere in the PT defined around literature based coordinates (MNI coordinates: x=-44 y=-27 z=13[6]). Finally, the PT ROI was defined as the overlap (logical AND) of the functional mask and the literature based sphere. A similar procedure was used for the vOT ROI. First, we created a functional mask, including the sum (logical OR) of the occipitotemporal activations across conditions (congruent and incongruent) and groups (normal and poor reading fluency) on a cluster-based family-wise error corrected (FWE-corr) threshold of $P<0.05$ (voxel-wise uncorrected $P<0.001$). Again, literature-based coordinates (MNI coordinates: x=-44 y=-57 z=-15[7]) were used to build a 12mm radius sphere in the vOT. After overlapping (logical AND) the functional mask and the vOT sphere, the ROI was further downsized (logical AND) by an anatomical mask of the fusiform gyrus based on the Talairach Daemon (TD) database[8] (WFU Pickatlas, version 2.4[9]).

Prediction analyses using general linear models: General linear models were calculated with SAS 9.4 (SAS Institute, Cary NC). Parameter estimates of linear regressions were calculated using the method of least squares as implemented in the PROC GLMSELECT process. To select independent variables with the highest predictive influence on the continuous dependent variable (reading fluency) a stepwise selection procedure was used. Independent variables with $P<0.15$ were entered and stayed in the regression model. To evaluate model fit of the model including the selected independent variables, adjusted R-square values were calculated. To avoid overfitting, a maximum of three factors were entered for selection into the model. To quantify the prediction error, predicted residuals sum of squares were cross-validated using the leave-one-out cross-validation method.

Supplementary Figures

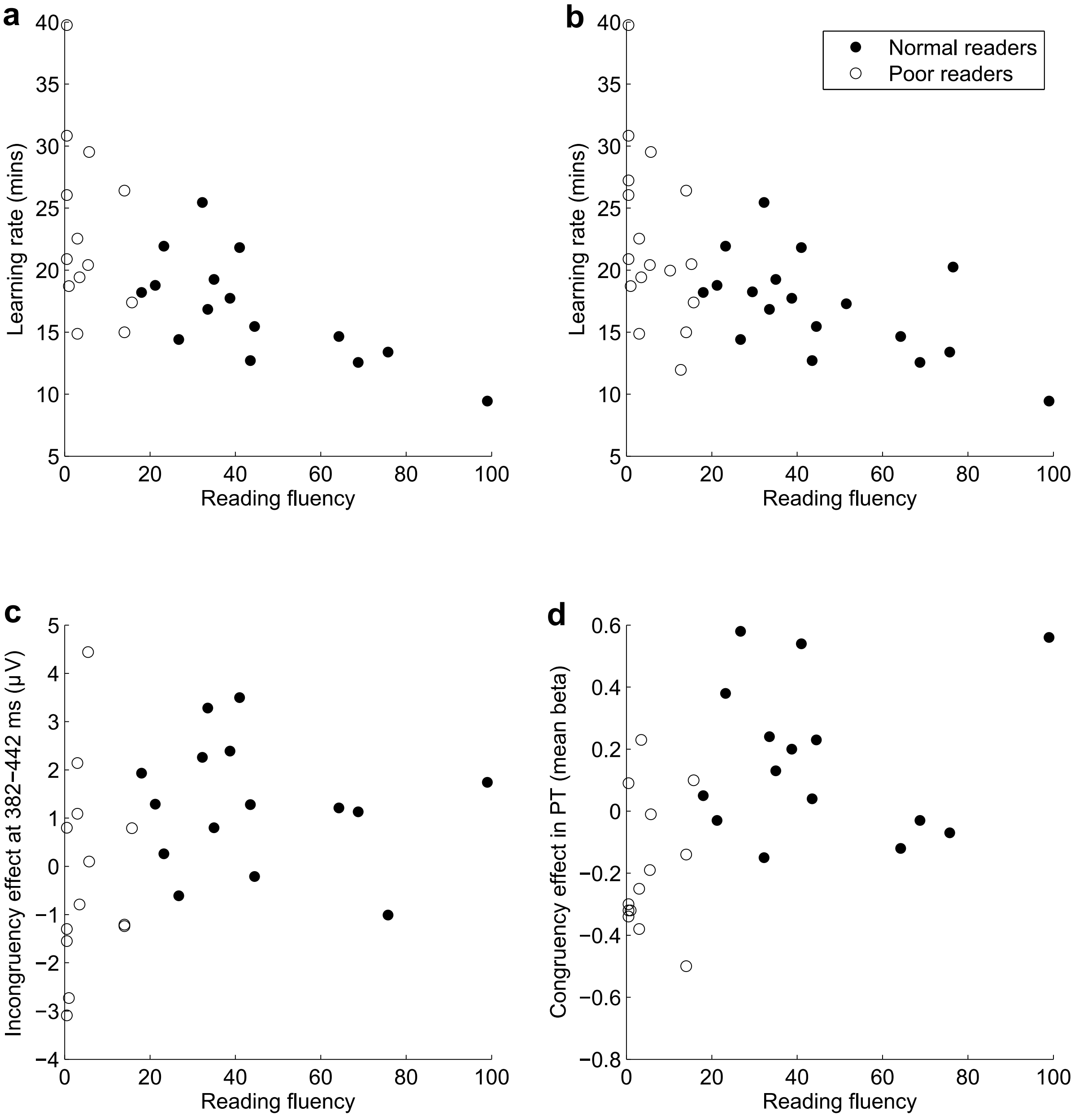

Supplementary Figure S1: Non-parametric correlations of prediction factors and mean reading fluency outcome. (a) Correlation within the main sample (*n*=28) of mean reading fluency and training duration, reflecting the learning rate in the artificial-letter training (*r*=-0.678, *P*=0.0017). (b) Correlation in the enlarged sample (*n*=35) of mean reading fluency and training duration, reflecting the learning rate in the artificial-letter training (*r*=-0.600, *P*=0.001). (c) Correlation of mean reading fluency and mean amplitude difference of incongruent and congruent trials at 382-442 ms over the left posterior electrodes of interest (*r*=0.437, *P*=0.057). (d) Correlation of mean reading fluency and mean difference of beta values during congruent compared to incongruent trials in the left planum temporale (PT) ROI (*r*=0.546, *P*=0.0153).

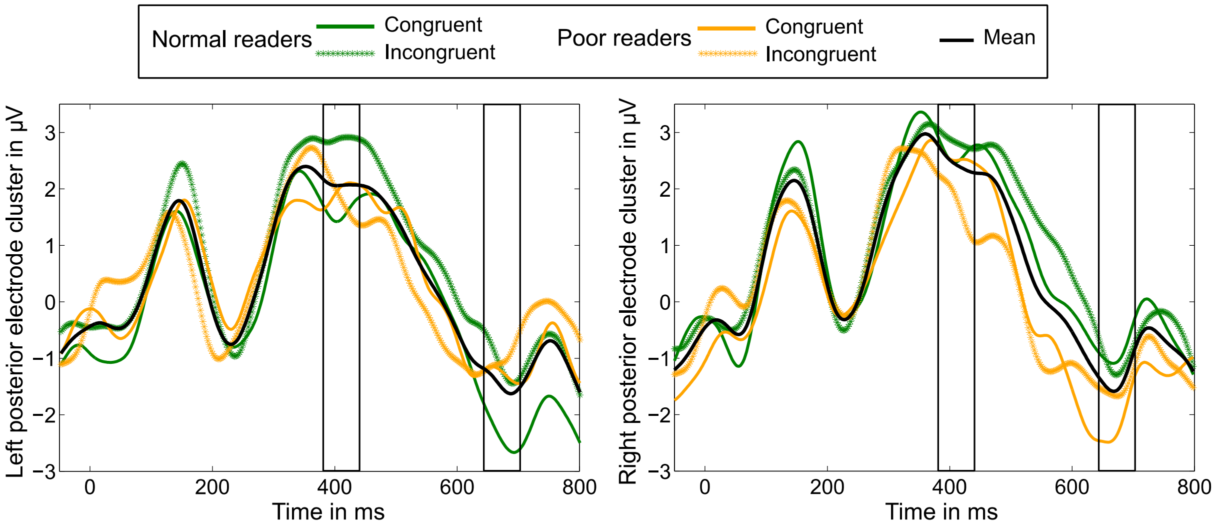

Supplementary Figure S2: ERP waves over the left and right posterior electrode clusters of interest are illustrated for each condition (congruent and incongruent) and reading group (normal and poor reading fluency). The marked segments represent the initial time window of audiovisual integration (382-442 ms) and the late negativity (644-704 ms).

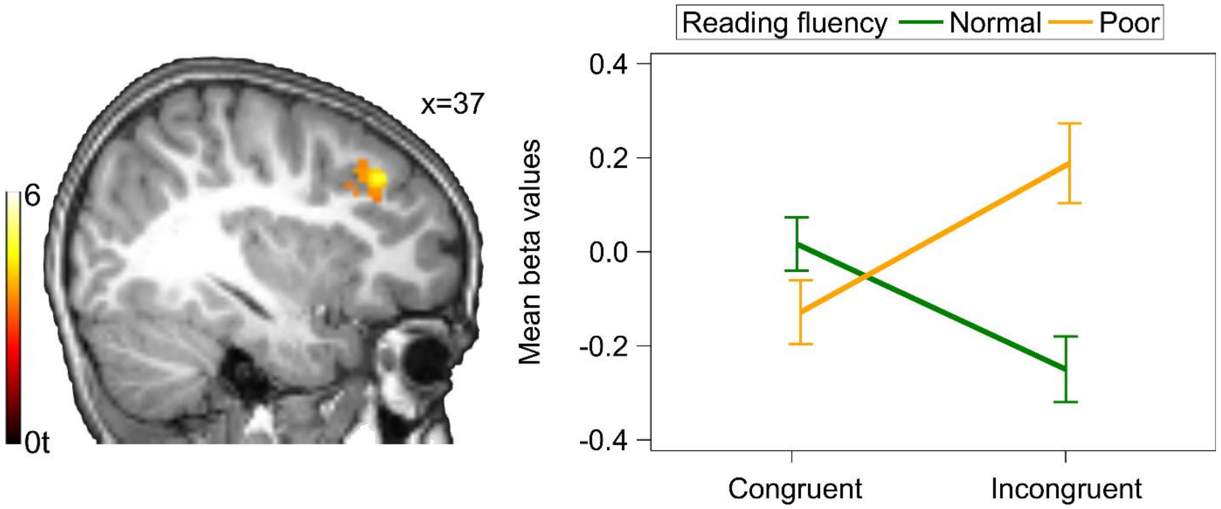

Supplementary Figure S3: Supplementary fMRI analyses. Whole brain analysis revealed a significant interaction of congruency and future reading fluency in the right MFG (peak MNI coordinates: x=37, y=33, z=39; k=63; T=4.77; cluster level family wise error corrected *P* =0.019 on a voxel-wise uncorrected level of *P*<0.001). Mean beta values were extracted from the brain region projected onto a pediatric structural T1 image normalized to MNI space. Error bars illustrate standard error of the mean.

Supplementary Tables

Supplementary Table S1: Complete sample of longitudinal study *n*=35

	Normal readers	Poor readers	Test statistic
Sex (female/male)	8/10	8/9	
Handedness (left/right)	3/15	0/17	
Familial risk for dyslexia[1]	0.52±0.18	0.51±0.12	*t*(33)=0.14, *P*=0.953
IQ estimate	108±14	100±14	*t*(33)=1.64, *P*=0.301
**Precursor skills of reading**			
Age in years T1	6.7±0.3	6.6±0.3	*t*(33)=0.76, *P*=0.564
Phonological awareness[2]	53.9±23.3	42.0±20.7	*t*(33)=1.60, *P*=0.301
RAN objects[2]	38.5±23.2	24.6±25.8	*t*(33)=1.67, *P*=0.301
letter-speech sound knowledge[3]	15.8±11.4	13.4±10.4	*t*(33)=0.64, *P*=0.608
Word reading[4]	3.6±4.8	2.2±3.0	*t*(33)=1.01, *P*=0.513
Non-word repetition[2]	37.0±23.0	29.5±23.2	*t*(33)=0.96, *P*=0.513
Passive vocabulary[2]	58.7±25.2	49.4±26.8	*t*(33)=1.06, *P*=0.513
Vocabulary – word meaning[2]	40.4±26.9	50.7±28.6	*t*(33)=-1.09, *P*=0.513
Training duration in minutes	17.1±3.9	22.4±6.9	*t*(33)=-2.81, *P*=0.041
Weighted training accuracy in %[5]	79.5±11.6	79.6±7.4	*t*(33)=-0.033, *P*=0.974
**Initial reading fluency skills**			
Age in years T2	7.3±0.2	7.3±0.3	*t*(33)=0.83, *P*=0.557
Word reading fluency[2]	49.6±23.7	6.8±7.4	*t*(33)=7.11, *P*<0.001
Pseudoword reading fluency[2]	41.8±24.2	5.7±7.2	*t*(33)=5.91, *P*<0.001

Values are mean ± standard deviation. *P*-values are FDR-adjusted. [1]highest parental ARHQ (adult reading history questionnaire) score; [2]percentile scores; [3]raw values; [4]number of correctly read one- or two-syllable upper case letter words out of 20; [5]trial-wise item-weighted accuracy; T1: prereading stage; T2: beginning reading stage

Supplementary Table S2: Non-parametric correlational analyses (*n*=28)

	Word reading fluency	Pseudoword reading fluency	Mean reading fluency
IQ estimate	0.166	0.088	0.145
Familial risk for dyslexia [1]	0.017	-0.079	-0.008
Phonological awareness[2]	0.358	0.309	0.354
RAN objects[2]	0.525*	0.617*	0.574*
Letter knowledge[3]	0.225	0.227	0.239
Non-word repetition[2]	0.209	0.099	0.171
Passive vocabulary[2]	0.322	0.204	0.266
Vocabulary-word meaning[2]	-0.275	-0.301	-0.283
Weighted training accuracy in % [4]	0.406$^{(*)}$	0.415$^{(*)}$	0.398$^{(*)}$
Training duration in minutes	-0.682*	-0.655*	-0.678*
ERP at 382-442 ms[5]	0.455$^{(*)}$	0.415$^{(*)}$	0.437$^{(*)}$
ERP at 644-704 ms[5]	0.366	0.157	0.333
Right MFG[6]	-0.533*	-0.406$^{(*)}$	-0.506*
Left PT ROI[6]	-0.543*	-0.457$^{(*)}$	-0.546*
Left vOT ROI: congruent[7]	0.441$^{(*)}$	0.386$^{(*)}$	0.457$^{(*)}$
Left vOT ROI: incongruent[7]	0.312	0.112	0.244
ERP modulation in right STG[6,8]	0.285	0.398$^{(*)}$	0.340

Correlation matrix with Spearman's *r* values: Values are mean ± standard deviation. Asterisks indicate values with significant FDR-corrected *P* values $*P<0.05$ $^{(*)}P<0.1$. [1]highest parental ARHQ score; [2]percentile scores; [3]raw values; [4]trial-wise item-weighted accuracy; [5]mean amplitude incongruency difference over left electrodes of interest; [6]incongruency difference of mean beta values, [7]mean beta values, [8]parametric modulation with mean amplitudes over left posterior electrodes of interest at 382-442 ms. ERP: event-related potential; MFG: middle frontal gyrus; PT: planum temporale; RAN: rapid automatized naming; STG: superior temporal gyrus; vOT: ventral occipitotemporal cortex

Supplementary Table S3: Supplementary multiple logistic regression models

Parameter	Maximum likelihood estimate	SE	Wald chi-square	*P*-value	Nagelkerke's pseudo R-square	Sensitivity	Specificity
**Enlarged sample *n*=35**							
**Behavioural model**							
Intercept	0.700	0.579	1.461	0.2267			
RAN[1]	-0.024	0.015	2.542	0.1109	0.104	64.7	50.0
Phonological awareness[1]				>0.15			
**Artificial-letter training**							
Intercept	-4.004	1.721	5.413	0.0200			
Learning rate[2]	0.204	0.088	5.324	0.0210	0.271	58.8	77.8
RAN[1]				>0.15			
**Main sample *n*=28**							
**Artificial-letter training & left-hemispheric incongruency difference of late negativity ERP (644-704 ms)**							
Intercept	-5.064	2.347	4.655	0.0310			
Learning rate[2]	0.287	0.129	4.953	0.0261	0.473	69.2	80.0
ERP (644-704 ms)[3]	-0.764	0.429	3.177	0.0747			
RAN[1]				>0.15			
**Artificial-letter training & left-hemispheric incongruency difference of initial ERP (382-442 ms) & late negativity ERP (644-704 ms)**							
Intercept	-4.780	2.411	3.931	0.0474			
Learning rate[2]	0.293	0.134	4.767	0.0290	0.571	69.2	73.3
ERP (644-704 ms)[3]	-0.789	0.446	3.130	0.0769			
ERP (382-442 ms)[3]	-0.524	0.317	2.728	0.0986			
**RAN & BOLD response in left vOT during congruent condition**							
Intercept	1.893	0.994	3.626	0.0569			
BOLD vOT[4]	-3.061	1.819	2.832	0.0924	0.343	61.5	66.7
RAN[1]	-0.035	0.023	2.245	0.1340			

[1]percentile scores; [2]training duration in minutes; [3]mean amplitude of incongruency difference over left posterior electrodes of interest; [4]mean beta values in the left vOT ROI; BOLD: blood-oxygen-level dependent, ERP: event-related potential, vOT: ventral occipitotemporal cortex, RAN: rapid automatized naming, SE: standard error.

Supplementary Table S4: General linear models (*n*=28)

Parameter	Estimate	SE	*t*-value	*P*-value	Adjusted R-square	Cross-validated PRESS
			**Behavioural model**			
Intercept	-7.137	9.958	-0.72	0.4802		
RAN[1]	0.499	0.209	2.38	0.0251	0.3264	14300
Phonological awareness[1]	0.371	0.196	1.89	0.0704		
			**Artificial-letter training**			
Intercept	52.224	19.231	2.72	0.0118		
Learning rate[2]	-2.045	0.662	-3.09	0.0049	0.4017	13387
Phonological awareness[1]	0.302	0.189	1.60	0.1216		
RAN[1]				>0.15		
			**Artificial-letter training & left-hemispheric incongruency difference of ERP mean amplitude (382-442 ms)**			
Intercept	75.741	12.797	5.92	<.0001		
Learning rate[2]	-2.503	0.615	-4.07	0.0004	0.3657	14105
ERP (382-442 ms)[3]				>0.15		
RAN[1]				>0.15		
			**Artificial-letter training & incongruency difference of BOLD response in left PT**			
Intercept	66.792	13.579	4.92	<.0001		
BOLD PT[4]	23.769	14.630	1.62	0.1168	0.4033	13447
Learning rate[2]	-2.060	0.656	-3.14	0.0043		
RAN[1]				>0.15		

[1]percentile scores; [2]training duration in minutes; [3]mean amplitude of incongruency difference over left posterior electrodes of interest at 382-442 ms; [4]mean beta values of incongruency difference in the left PT ROI; BOLD: blood-oxygen-level dependent, ERP: event-related potential, PT: planum temporale, RAN: rapid automatized naming, SE: standard error, PRESS: predicted residuals sum of squares.

References

1. Brandeis, D. & Lehmann, D. Event-related potentials of the brain and cognitive processes: approaches and applications. *Neuropsychologia* **24,** 151-168 (1986).

2. Hoeft, F., *et al.* Prediction of children's reading skills using behavioral, functional, and structural neuroimaging measures. *Behav. Neurosci.* **121,** 602-613 (2007).

3. Allen, P.J., Josephs, O. & Turner, R. A method for removing imaging artifact from continuous EEG recorded during functional MRI. *Neuroimage* **12,** 230-239 (2000).

4. Wilke, M., Holland, S.K., Altaye, M. & Gaser, C. Template-O-Matic: a toolbox for creating customized pediatric templates. *Neuroimage* **41,** 903-913 (2008).

5. Mazaika, P., Whitfield-Gabrieli, S., Reiss, A. & Glover, G. Artifact repair for fMRI data from high motion clinical subjects. in *Organization of Human Brain Mapping International Conference* (Chicago, IL, 2007).

6. Blau, V., *et al.* Deviant processing of letters and speech sounds as proximate cause of reading failure: a functional magnetic resonance imaging study of dyslexic children. *Brain* **133,** 868-879 (2010).

7. Vandermosten, M., Hoeft, F. & Norton, E.S. Integrating MRI brain imaging studies of pre-reading children with current theories of developmental dyslexia: a review and quantitative meta-analysis. *Current Opinion in Behavioral Sciences* **10,** 155-161 (2016).

8. Lancaster, J.L., *et al.* Automated Talairach atlas labels for functional brain mapping. *Hum. Brain Mapp.* **10,** 120-131 (2000).

9. Maldjian, J.A., Laurienti, P.J., Kraft, R.A. & Burdette, J.H. An automated method for neuroanatomic and cytoarchitectonic atlas-based interrogation of fMRI data sets. *Neuroimage* **19,** 1233-1239 (2003).
